# Effects of maternal consumption of morphine on rat skeletal system development

**DOI:** 10.1186/s12891-021-04321-6

**Published:** 2021-05-13

**Authors:** Maryam Saeidinezhad, Vahid Razban, Hosein Safizadeh, Massood Ezzatabadipour

**Affiliations:** 1 Neuroscience Research Center, Institute of Neuropharmacology, Kerman University of Medical Sciences, Somayeh Cross-road, Sajad Boulevard, Ebnesina Street, Kerman, 7619813159 Iran; 2 Department of Anatomical Sciences, School of Medicine, Kerman University of Medical Sciences, Kerman, Iran; 3 Department of Molecular Medicine, School of Advanced Medical Sciences and Technologies, Shiraz University of Medical Sciences, Shiraz, Iran; 4 Stem cell Technology Research Center, Shiraz University of Medical Sciences, Shiraz, Iran

**Keywords:** Morphine dependency, Skeletal system development, Ossification center, Growth plate

## Abstract

**Background:**

Opioid abuse is among the most ubiquitous issues world-wide, and when it happens in mothers, it puts them at risk of diseases that can be transferred to the next generation. Previous studies have indicated that morphine addiction during pregnancy could inhibit development in rat embryos and infants.

The present study focused on the effects of maternal consumption of morphine on rat skeletal system development and also investigate the molecular pathway of chondrogenesis and osteogenesis of infants from control and addicted rat groups.

**Methods:**

Thirty-two female rats were randomly assigned to four groups. The groups consisted of one- and seven-day-old female infants which were born of morphine-dependent mothers and a control group for each of them. Experimental groups received oral morphine at the final dose of 0.4 mg/ml/day. Withdrawal signs were confirmation of morphine dependency. Female rats were crossed with male rats and coupling time was recorded. Fixed bones of all groups were processed and then stained by hematoxyline-eosin method. Thickness and cell number of proximal and distal growth plate of bones were measured. The cartilage and bone cells were stained by alcian blue/alizarin red method. Additionally, the gene expression of alkaline phosphatase, osteocalcin, and COLL2 and SOX9 gene expression were studied immuno-histochemically.

**Results:**

Unfavorable effects of morphine on histological measurements were observed in one-day and seven-day infants, with more effects on seven-day infants. The thickness and cell number of the proximal and distal growth plate of morphine-dependent rat offsprings were reduced significantly. Furthermore, morphine reduced growth of primary and secondary ossification centers, and thus, longitudinal bone growth was reduced. Moreover, a decrease in the alkaline phosphatase, osteocalcin, COLL2 and SOX9 gene expression, and the number of stained cells was observed. More adverse effects of morphine in seven-day infants compared to one-day infants which showed the time dependent of morphine to the time length of administration.

**Conclusion:**

Histochemistry and immunohistochemistry findings on cartilage and bone matrix formation, as well as protein expression of chondrogenic and osteogenic markers suggest that morphine dependence in pregnant mothers may impair intra-cartilaginous osteogenesis in post-natal rats.

**Supplementary Information:**

The online version contains supplementary material available at 10.1186/s12891-021-04321-6.

## Background

The skeletal system development begins in the embryonic period of the intrauterine life with the formation of primary ossification centers in the middle of long bone shafts and is followed by the appearance of the secondary ossification centers in the epiphyses of long bones in the postnatal period [1]. Longitudinal bone growth falls out through the endochondral ossification process and within the proximal and distal growth plate cartilages, which remain functional until the proximodistal elongation stops at the opposite ends of the shaft of each long bone [2]. Cells in the growth plate cartilages lengthen the long bones during the five successive stages: Resting (Reserve), Proliferating, Hypertrophy, Calcifying and Ossification [3]. Endochondral ossification starts with the migration and condensation of the mesenchymal cells. Then these osteochondrogenic cells are differentiated into chondroblasts, which are known as the prime key of endochondral bone formation [4]; therefore, the shaft of long bones is formed in the intrauterine period. Reports on in vitro as well as prenatal and postnatal skeletogenesis have provided evidence on the effect of various factors such as cell migration and differentiation oxygen tension and angiogenesis, and hormone existence and calcium-based mineral supply. These factors exert their effects on osteogenesis and chondrogenesis mainly through gene expression modifications during mesenchymal differentiation which has been shown to be responsive to environmental signals [4–8]. Considering the essential role of these factors in bone and cartilage formation and differentiation, these complex processes may be disturbed by the alteration of any of these factors directly or indirectly through the interference of teratogenic agents at the critical time. On the basis of present evidence, opium and its most important alkaloid, i.e., morphine, are among teratogenic agents. An association between congenital defects and prescription drugs or opioid abuse has been reported [9]. The effect of morphine on osteogenesis is conducted by the mu-opioid receptors [10]. The presence of opioid receptors on embryonic and mesenchymal stem cells, chondroblasts and chondrocytes, allows the intervention of endogenous and exogenous opioids in cell functions. Results of our previous work indicated that morphine prevents mesenchymal stem cell proliferation, phenotype, and differentiation in addition to reduction in cell proliferation of the growth plate and its thickness [11, 12]. Significant reduction of rat ovarian steroids after administration of morphine during pregnancy, amniotic fluid reduction [13], and developmental delay of different parts of the placenta and fetus have been observed [14]. Disruption of the ovarian cycle, cessation of spermatogenesis, and death of a number of fetuses after morphine abuse during pregnancy have also been reported [15]. Gradual passage of morphine through the placenta [16], quick diffusion into the various tissues [17], and reduction of placenta weight and diameter and fetus weight have already been studied. Teratogenic effects of morphine in rats take place mostly in the organogenesis period (2nd week) of prenatal development [18]. Different studies have also documented the adverse effect of morphine on the regenerative capacity of stem cells and the impaired healing of wounds and bone injuries [19, 20]. Women’s substance abuse is worthy of attention from different points of view including anatomical and physiological characteristics, the issue of pregnancy and the health of the infant, and the social and child-rearing role of mothers. Literature review does not reveal accurate statistics on women’s dependence on drugs, according to the United Nation’s annual reports on drug use, although licit and illicit use of opiates is third after the use of cannabis and amphetamines, it poses a greater and more severe health risk, and women are more likely to suffer from the consequences of drug use than men are [21].

As for these facts, the direct or indirect effects of morphine on chondrocytes and mesenchymal cells, and the participation of these cells in bone and cartilage formation, we attempted to evaluate the impact of morphine consumption during pregnancy on bone development and primary and secondary ossification center formation in the pre- and post-natal period.

## Methods

### Animals

This research was carried out on 48 healthy 6- to 8-week-old (weighing about 200 g) female Wistar rats. The animals were purchased from the animal house of Afzalipour School of Medicine, Kerman, Iran. The research was conducted with the approval of the ethics committee of Kerman University of Medical Sciences (approval number: IR.KMU.REC.1393,74). Animals were randomly allocated to two equal morphine-dependent and control groups and were kept in 12 cages at 21–23 °C, 12 h light/darkness cycles and with free access to water, rodent chow and ad libitum feeding [22, 23]. All sections of the present study adhere to the “ARRIVE guidelines” for reporting in vivo experiments in animal research. Completed “ARRIVE guidelines” checklist is included in Supplementary 1.

### Morphine dependency and mating

Morphine dependence treatment was conducted before pregnancy. Morphine was dissolved in the drinking water of animals. To eliminate the bitter taste of morphine 2 g/l sucrose was added to the water. The control group (24 female rats) received water only. To make the animals addicted, morphine concentration was gradually increased as follows: 0.1 mg/ml on the first and second days, 0.2 mg/ml on the third and fourth days, 0.3 mg/ml on the fifth and sixth days and 0.4 mg/ml until the fifteenth day. The final dose (0.4 mg/ml) was continued until the end of pregnancy and then during lactation. To ensure morphine dependency, 2 mg/kg naloxone was injected intraperitoneally in one randomly selected animal from the morphine-dependent group, and then withdrawal signs were evaluated [24, 25]. Both control and morphine-dependent females were exposed to healthy male rats individually. After 24 h, the rats with vaginal plaque were identified and the coupling time was determined as embryonic day 0 (E0).

### Fixation and tissue processing

The pregnant animals in each of the control and morphine-dependent groups were allowed to deliver their newborns at day 21 of gestation. Eight infants were randomly selected [26] from each of the one and seven-day infants and then their posterior limbs were carefully separated. Their femur bones were disarticulated and soft tissues were removed in all groups. The infants were euthanized by cervical dislocation. In order to fixate bones for alcian blue and alizarin red staining, they were fixed in 10% formalin solution for 48 h with one formalin replacement after the first day of fixation. Samples from day one and day seven infants were kept under 10% nitric solutions for 0.5 and 1 h, respectively before tissue processing. Preparation of tissue samples using an automatic tissue processor was conducted in the following order: dehydration, clearing, embedding and blocking. Processed paraffin-embedded samples were cut by microtome into 5-μm sections [27, 28].

### Staining

For histological studies, samples were stained by hematoxyline and eosin (H&E) [29, 30] to detect the growth plate cartilage in both proximal and distal ends. Thickness of growth plate cartilages was measured and photographed. Cell density in proliferative and hypertrophy zones of growth plate cartilage in all groups were counted in all samples. Alcian blue and alizarin red [31, 32] were used for specific staining of cartilage and bone tissues, respectively. Comparison and evaluation of chondrogenesis and osteogenesis were performed based on a spectrophotometric assay by extracting and quantifying the amount of stain absorbed by the sections in previous experiments. Alizarin red stain was washed by 15 min` exposure to a solution containing 10% acetyl pyridinium chloride in a 10 mM sodium phosphate buffer (pH 7.0). The optical density (OD) of the extracted stains was measured at 562 nm. Alcian-blue-stained sections were dissolved in 1% sodium dodecyl sulfate and the OD was read at 605 nm. The ODs for alizarin red and alcian blue are proportional to the amount of calcium deposition and glycosaminoglycan (GAG) contents, respectively. The optical densities were normalized related to the area of stained tissue.

### Immunohistochemistry

Expression of late and early chondrogenic and osteogenic specific markers were surveyed by immuno-histochemistry (IHC). Antibodies against alkaline phosphatase (ALP) from Abcam company (Cat# ab65834) and osteocalcin (OCN) from Millipore company (Cat# ab10911), as osteogenesis markers, and collagen type 2 (COLL2) from Abcam company (Cat# ab34712) and SRY-box transcription factor 9 (SOX9) from Abcam company (Cat# ab3697), as chondrogenic markers, were purchased from Abcam and Millipore companies [22, 33]. In IHC cell’s nuclei were stained by hematoxyline.

### Statistical analysis

Sample size (for each of four groups, *n* = 8) was almost based on a similar previous study [26]. The number of offspring in the control and morphine-dependent groups was 78 and 43 respectively. The infants` bone samples of four groups were randomly selected and, statistical analysis was conducted randomly on 6 out of 8. Data analysis was performed using SPSS software version 16. Semi-quantitively determination of gene expression of alkaline phosphatase, osteocalcin, SOX9 and COLL2 was done using ImageJ and prism software [34]. Data are presented as mean ± SEM. T-test was used to evaluate the intergroup differences; *P* value< 0.05 was considered significant.

## Results

Since the development of ossification centers in rats lasts up to the seventh day after birth, this study was conducted on days one and seven after birth [11]. The highest rate of longitudinal growth of long bone occurs in the intrauterine stage, [35] which was one of the reasons we concentrated on this period. Chondrocytes, as master regulatory cells, fuel bone growth through the hypertrophy process [36]. These led us to focus on what happen to chondrocytes during endochondral ossification. In the present study, the effect of morphine on the proximal and distal growth plate cartilages of the femur has been surveyed and compared based on the thickness of their five zones` (reserve, proliferation, hypertrophy, differentiation and calcification) and the cell numbers between morphine-dependent and control neonates. In general, the distal growth plate cartilage of the femur is the growing end and thicker than the proximal growth plate.

### Effect of morphine on thickness of proximal growth plate cartilage

**Fig. 1:**
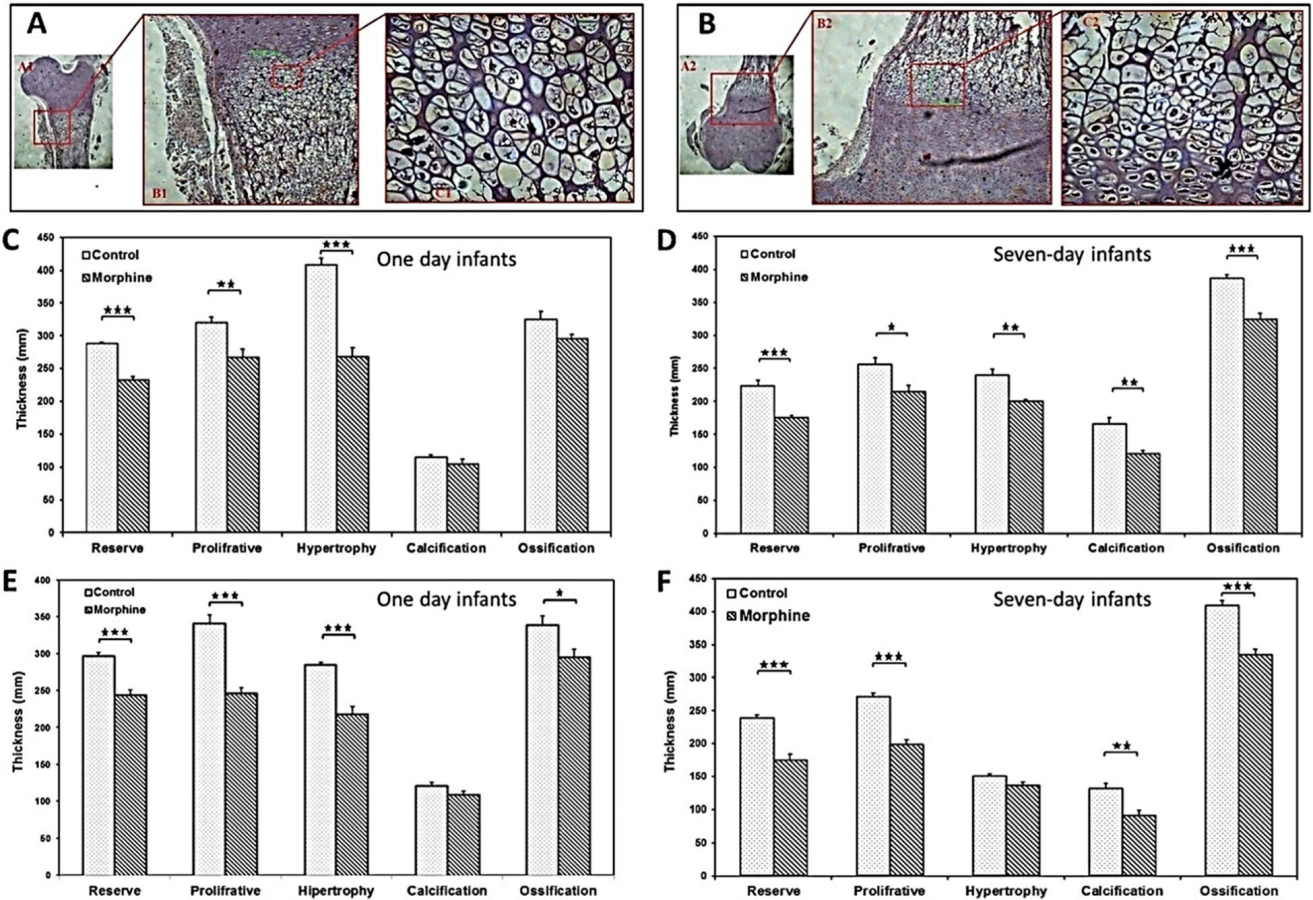
Hematoxylin and Eosin staining: micrograph shows the proximal (**a**) and distal (**b**) growth plate cartilage. Comparison of the five zones thickness of the proximal (**c-d**) and distal (**e-f**) growth plate cartilage between morphine-dependent and control groups. *n* = 8. Bars are representative of the SEM of samples (*P < 0.05, **P < 0.01, ***P < 0.001)

The results summarized in Fig. 1a-b demonstrated a significant difference in the size of reserve (*p* < 0.001), proliferative (*p* < 0.01) and hypertrophic (*p* < 0.001) zones between the morphine-dependent group and the control group of one-day infants. Furthermore, the total growth plate thickness significantly decreased in the morphine-dependent group. In addition to the significant decrease of total thickness in the morphine-dependent group in comparison with the control group of seven-day infants, the thickness of each of the reserve (*p* < 0.001), proliferative (*p* < 0.05), hypertrophic (*p* < 0.01), calcification (*p* < 0.01) and ossification (*p* < 0.001) zones shows a significant difference between morphine-dependent and control groups.

### Effect of morphine on thickness of distal growth plate cartilage

As shown in Fig. 1c-d, the thickness of four out of the reserve (*p* < 0.001), proliferative (*p* < 0.001), hypertrophy (*p* < 0.001) and ossification (*p* < 0.05) zones were significantly decreased in the morphine-dependent group compared to control group in one-day infants. The same reduction in thickness were also observed in reserve (*p* < 0.001), proliferative (*p* < 0.001), calcification(*p* < 0.01) and ossification (*p* < 0.001) zones of seven-days infants, as well. Moreover, the total growth plate thickness decreased significantly in the morphine-dependent group compared to control group.

### Effect of morphine on cell number

**Fig. 2:**
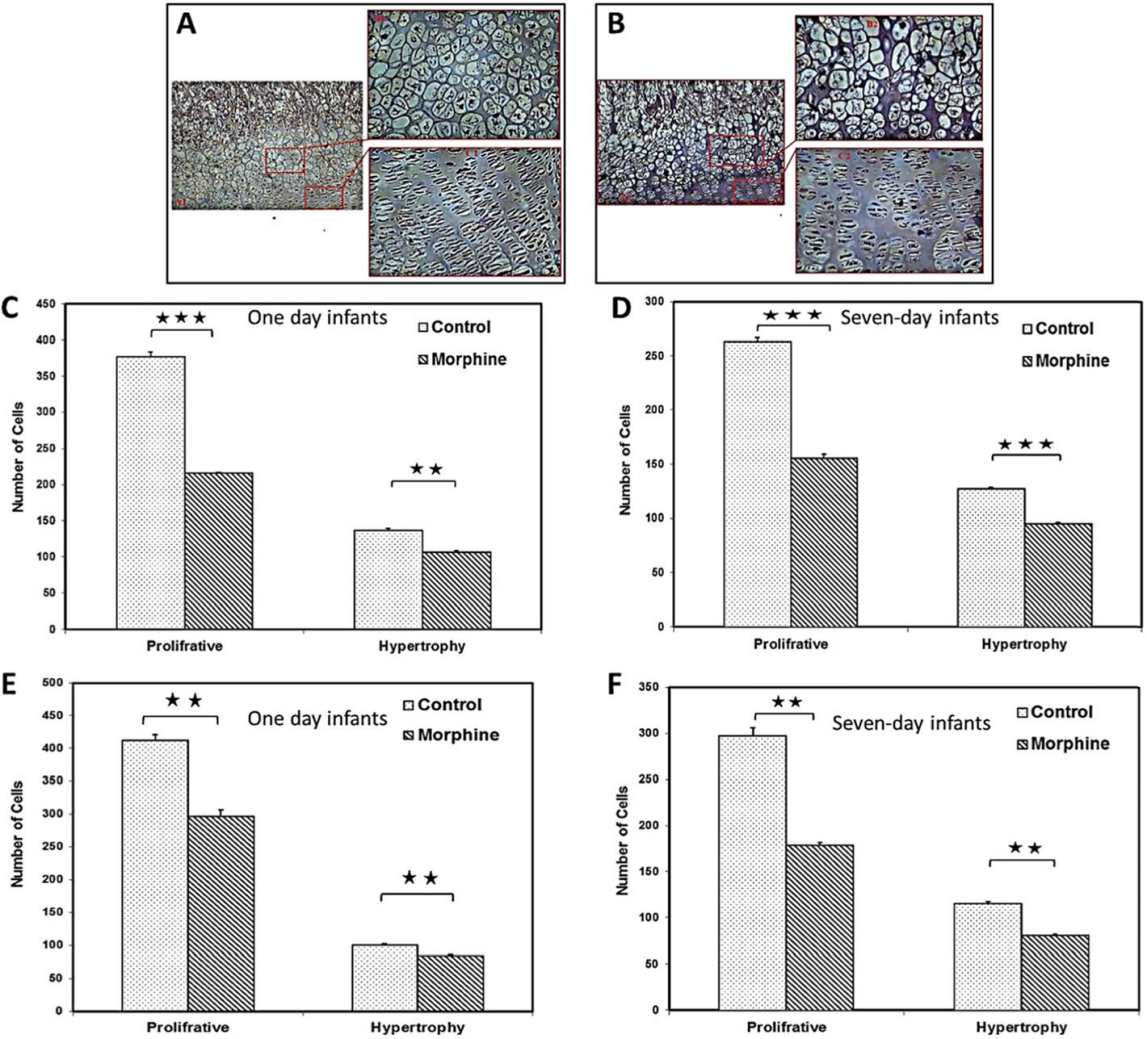
Hematoxylin and Eosin staining: micrograph displays the proliferative and hypertrophic zones in control (**a**) and morphine-dependent (**b**) groups. Cell number comparison of proliferative and hypertrophic zones of the proximal (**c-d**) and distal (**e-f**) growth plate cartilage between morphine-dependent and control groups. Bars are representative of the SEM of samples. *P < 0.05, **P < 0.01, ***P < 0.001. *n* = 8

Based on data presented in Fig. 2, morphine consumption significantly reduced the number of cells in proliferative (*p* < 0.001) and hypertrophic (*p* < 0.01) zones of proximal and proliferative (*p* < 0.001) and hypertrophic (*p* < 0.001) zones of distal growth plate cartilage in one-day infants. The same results were observed in proliferative (*p* < 0.01) and hypertrophic (*p* < 0.01) zones of proximal and proliferative (*p* < 0.01) and hypertrophic (*p* < 0.01) zones of distal growth plate cartilage in seven-day infants.

### Results of alcian blue and alizarin red staining

Cartilaginous mold formation and ossification, as preliminary steps of bone formation, were also investigated by alcian blue and alizarin red staining, respectively.

**Fig. 3:**
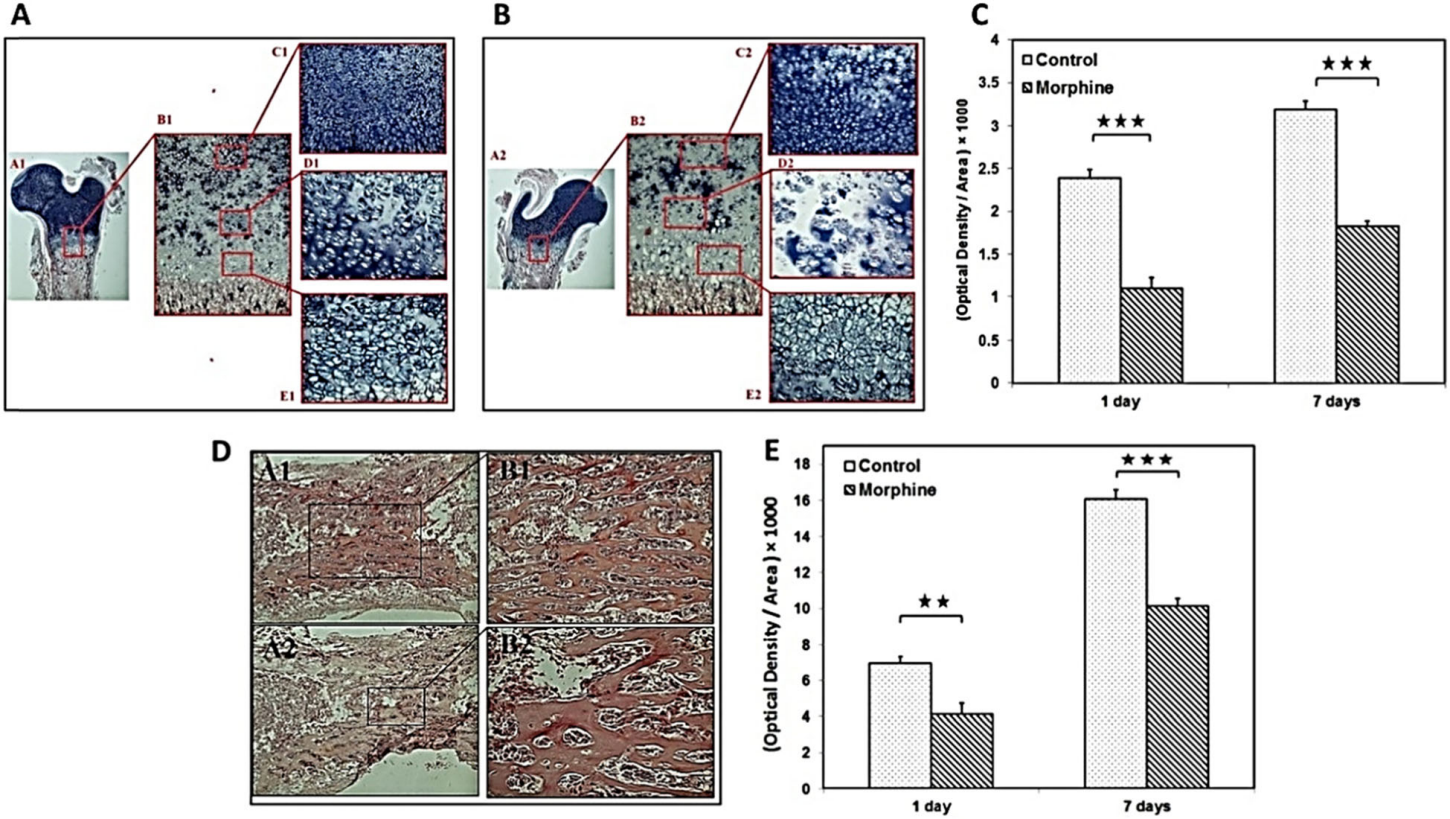
Alcian blue staining: The bone metaphysis in the control (**a**) and morphine-dependent (**b**) groups (B1 and B2 show different zones of the growth cartilage, 40 X). Comparison of the optical density of Alcian blue /Area between (represented the GAG contents) two groups in 1 day and seven-day infants (**c**). Alizarin red staining: The bone diaphysis in the control (**d**; A1, B1) and morphine-dependent groups (**d**; A2, B2) (40X). *n* = 8. Comparison of the optical density of Alizarin red /Area (represented the calcium deposition) between two groups in 1 day and seven-day infants (**e**)

Significant reduction of cell density was clearly observable in all growth plate cartilage zones of the morphine-dependent group in comparison with the control group after alcian blue staining (Fig. 3a-b). Furthermore, the results show that GAG contents significantly decreased in the morphine-dependent group (*p* < 0.001) in both one-day and seven-day infants (Fig. 3c). Images of the bone diaphysis in the morphine-dependent and control groups after staining by alizarin red are demonstrated in Fig. 3d. Moreover, the results show that GAG contents significantly decreased in the morphine-dependent group in one-day (*p* < 0.01) and seven-day (*p* < 0.001) infants (Fig. 3e). Cell density was reduced in the morphine-dependent group for one and seven-day infants compared to the controls. Additionally, calcium deposition was significantly decreased in comparison with the control groups.

### Effect of morphine on alkaline phosphatase and osteocalcin expression

Expression of SOX9 (essential transcription factor for initiation of chondrocyte differentiation from condensed mesenchymal cells) and COLL2 (main hyaline cartilage collagen type produced by chondrocytes) as early and late markers for cartilage tissue formation, respectively, and alkaline phosphatase and osteocalcin (as early and late osteogenesis and bone formation markers, respectively) [4, 37] were also assessed so as to shed light on the mechanistic processes by which morphine affects the cartilage and bone formation. Mesenchymal stem cell conversion to chondroblasts is done in four sequential steps [38]. SOX9 and Coll2 were expressed in the second and final steps of this process. Down-regulation of SOX9 in the hypertrophic zone of growth plate might be an essential step to allow vascular invasion and endochondral ossification [39].

**Fig. 4:**
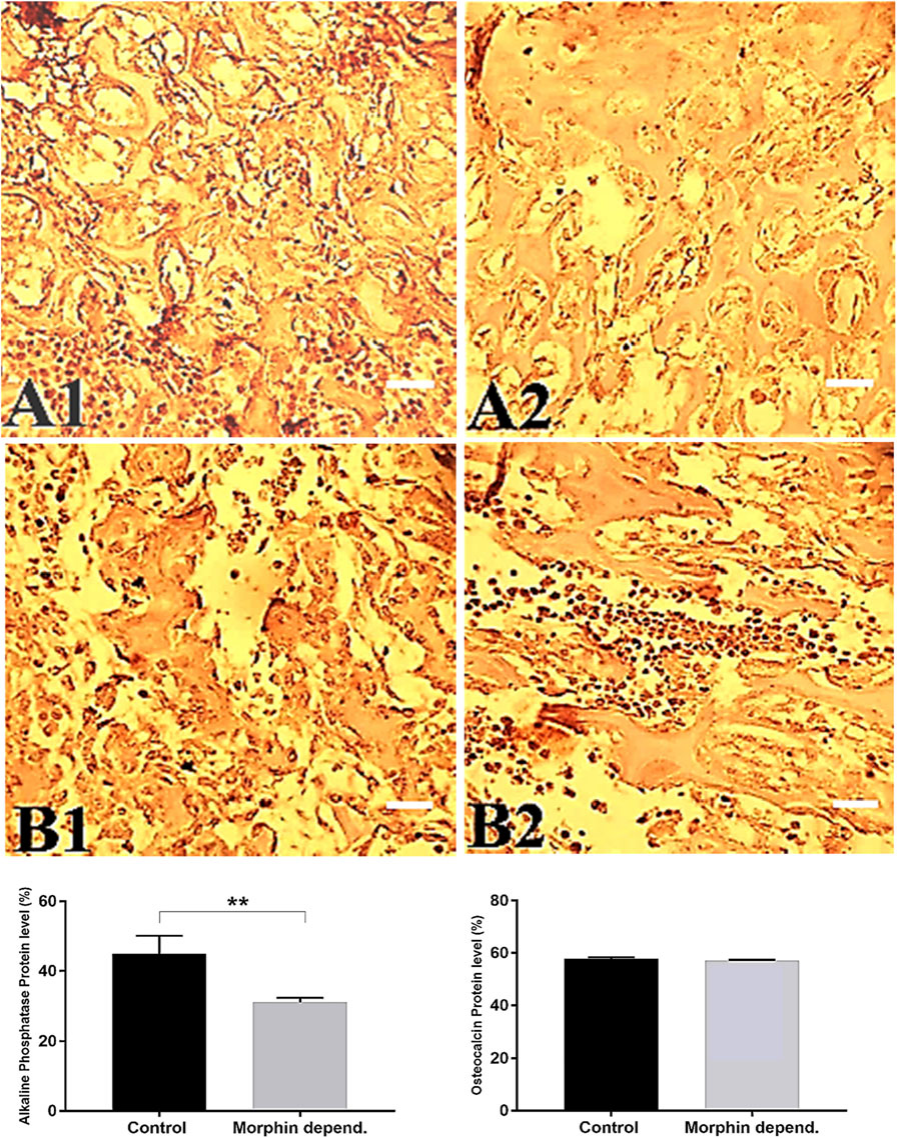
Alkaline phosphatase gene expression in the bone diaphysis of the control (A1) and morphine-dependent (A2) groups. Osteocalcin gene expression in the bone diaphysis of the control (B1) and morphine-dependent (B2) groups (40 X). More intense staining’s are detectable in A1 and B1 compared to A2 and B2, respectively. Cell’s nuclei were stained by hematoxylin. Scale bar = 10 μm. The graphs show the gene expression levels of alkaline phosphatase and osteocalcin between the control and morphine-dependent groups. PR means proliferative region; R means reserve region; depend. is abbreviated form of dependent

Comparison of gene expression of alkaline phosphatase and osteocalcin in growth plate cartilage cells between morphine-dependent and control groups are demonstrated in Fig. 4; Especially for ALP, it seems that a decrease in the gene expression and the number of stained cells has occurred. A semi-quantitative assay (its results are shown in the graph inside the Fig. 4) was also conducted in order to find out the differences between the data (A1 & A2; B1 & B2).

### Effect of morphine on expression of SOX9 and COLL2

**Fig. 5:**
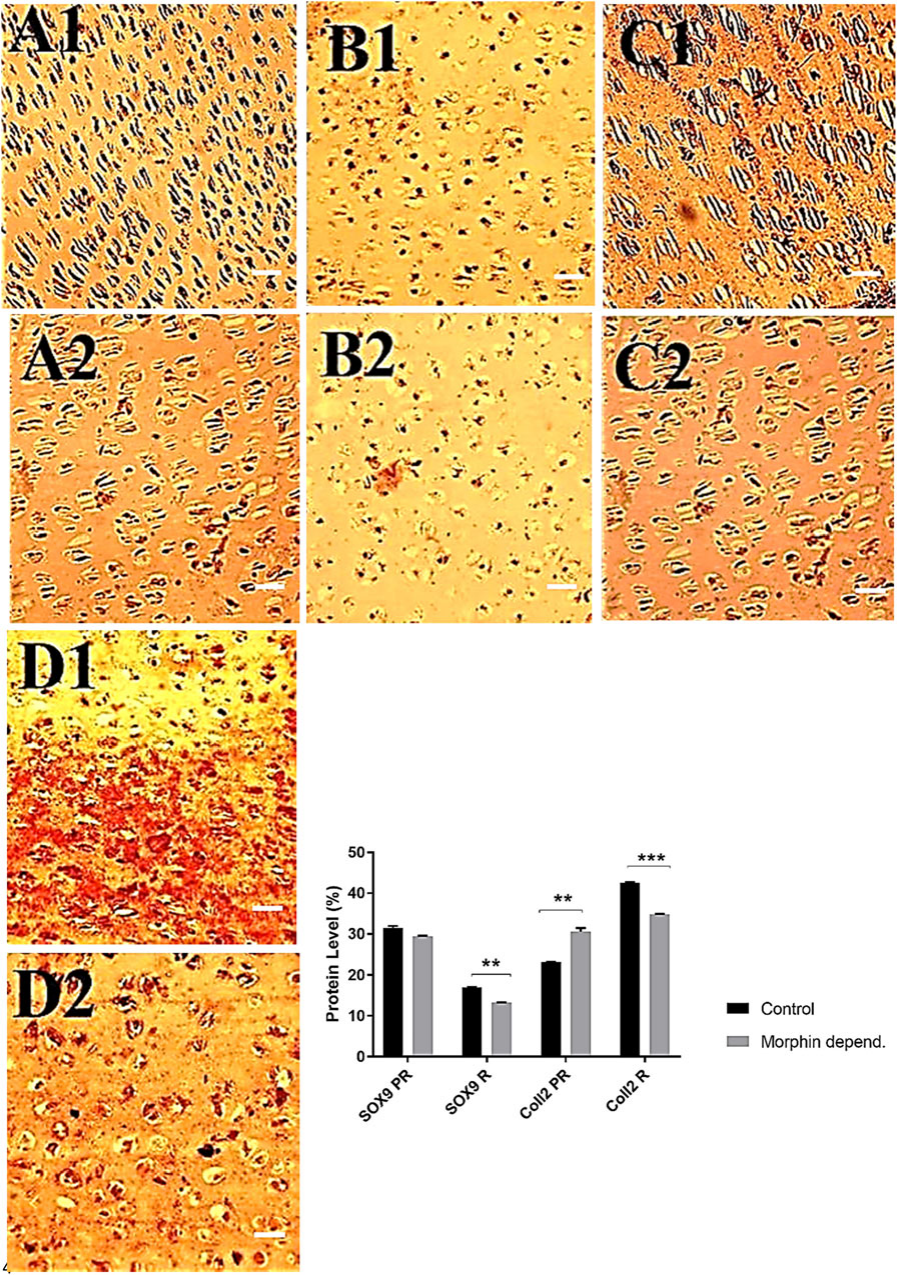
The stained cells by anti SOX9 antibody in the proliferative region of the control (A1) and morphine-dependent (A2) groups. The stained cells for SOX9 in the reserve region of the control (B1) and morphine-dependent (B2) groups. The stained cells by antiColl2 antibody in the proliferative region of the control (C1) and morphine-dependent (C2) groups. The stained cells for Coll2 in the reserve region of the control (D1) and morphine-dependent (D2) groups (40 X). More intense staining’s are detectable in B1, and D1 compared to B2, and D2, respectively. Cell’s nuclei were stained by hematoxyline. Scale bar = 10 μm. The graph shows the difference in the gene expression of SOX9 and Coll2 between the control and morphine-dependent groups. PR means proliferative region; R means reserve region & depend. is abbreviated form of dependent

Comparison of gene expression of SOX9 and COLL2 in growth plate cartilage cells between the morphine-dependent and control groups is demonstrated in Fig. 5. It seems that a decrease in the gene expression and the number of stained cells has occurred. The results of a semi-quantative survey revealed the differences between the data (A1 & A2; B1 & B2; C1 & C2; D1 & D2) are shown in a graph inside the Fig. 5. This data evaluation showed that the gene expression of COLL2 in the proliferative region was higher in the morphine-dependent infants than controls, while this value was lower in the reserve region in the morphine-dependent infants than controls.

## Discussion

Cartilaginous formation of long bone and ossification centers are complex processes that are affected by many factors. In addition to genetic, nutritional and environmental factors, hormones and many cytokines could affect longitudinal bone growth [4, 40, 41]. Morphine, which was first isolated in 1805 from opium [42] and used as a strong analgesic, has controversial (proliferative and apoptotic) effects on different cells [43–46]. In a previous study, we made inquiries about the effect of morphine on growth plate cartilage [11]. In the present study, the impact of maternal morphine consumption on the formation of long bones and ossification centers and growth plate cartilage maturity was investigated.

In brief, our results confirmed harmful effects of postnatal morphine consumption on bone formation and bone length. Although what was done in the present study has not been done so far, our results are consistent with the results of previous studies [11, 47, 48]. Significant difference of proximal and distal growth plate cartilage thickness between morphine-dependent and control groups both in one and seven-day infants revealed the adverse or maybe apoptotic effect of morphine on chondrocytes. Significant decrease of cell density in the morphine-dependent group after alizarin red staining and significant decrease of cell number in proliferative and hypertrophic zones could be due to decreased cell viability and/or proliferation, although more specific tests are necessary to validate this. These results are in accordance to the previous studies [11]. The induction of chondrocyte apoptosis or inhibition of cell proliferation by morphine and its mechanism in addicted subjects during embryo development could be an open area for future research, as there are reports on cytotoxic effects of morphine on chondrocytes in vitro after short time exposure to opioid or in vivo when it is administered as an analgesic [49–51]. Collagen 2 (COLL2) showed decreased expression in proliferative region of control group compared to the addicted group (graph inside of the Fig. 5), however, higher expression of this protein in the reserve region was more noticeable. Moreover, higher production of matrix, detected by GAG and calcium staining, thicker tissues and larger size of bones and cartilages in controls compared to addicted groups implies the adverse effects morphine on skeletal system.

Based on our data, the decreased COLL2 detection in the cartilage matrix by immunohistochemistry experiments may explain, in part, the reduced number of cells in the morphine-dependent group. On the other hand, COLL2 is a direct target of the down-stream gene SOX9; hence, the decline of this master transcription factor of chondrocyte differentiation, particularly in reserve region, may also be involved in defective cartilage matrix formation. Decreased staining of osteocalcin and especially alkaline phosphatase protein expression in immunohistochemistry indicates that bone formation has been disturbed by morphine i.e., morphine could exert its harmful effect on the cartilaginous bone formation through inhibiting the expression of SOX9 and its down-stream gene, COLL2, which would lead to the disabling of the “motor engine” of bone growth. Chondrocyte proliferation, vertical column cell alignment and differentiation of proliferating to prehypertrophic and hypertrophic chondrocytes, which leads to ten-fold cell volume increase are important primitive events that occur in cartilage-bone transition in endochondral bone formation [39]. SOX9, as a transcription factor, is highly expressed in chondroprogenitor, proliferating and prehypertrophic chondrocytes of the fetal growth plate [39]. It seems that influence of morphine upon its receptors on the chondrocytes could explain the remarkable drop-off in the expression of these genes and the impairment of cartilage-bone transition. The dose-dependent apoptotic effect of morphine on various cell types such as chondrocytes has been reported before [52]. In line with the results of our previous report [11], the data of the present study also indicated that the time length of morphine administration intensifies its effects on chondrocytes as more zones of the growth plates in seven-day infants were affected compared with those of one-day infants. As the highest rate of longitudinal growth of long bone starts in the fetal period [35], the detrimental effects of morphine are expected to be more important at this stage.

Strong connection between angiogenesis and osteogenesis [53], molecular pathway which couples these two processes [54] and inhibition of bone recovery and development by anti-angiogenic drugs [55] and opioid administration [20] could mean that morphine indirectly affects bone growth through angiogenesis [19]. However, this suggestion needs more investigations to be validated and controversial report is also available [56]. The other pathway which might be affected by morphine dependency could be the reported adverse effect of opioids on osteocalcin synthesis of osteoblasts [57].

Growth plates in rats stay open for a long period after puberty and perhaps throughout the normal life of the animal [58]. Evaluating the results of a study on cartilage growth showed that morphine dependence in male rats causes significant decrease in proliferation region cells and a significant reduction in the thickness of the growth cartilage. This change was more evident in rats with a longer duration of dependence. But morphine did not change the number of hypertrophy region cells in the growth plate [14]. These results suggest that morphine can have an inhibitory effect on the growth of cartilage cells.

According to the results of Roach et al. the reduction of the thickness of cartilage growth in the final weeks of activity is due to reduction in the number and size of hypertrophy cells [59]. Osteogenesis is an aerobic process and to continue this process, angiogenesis is necessary, and given that morphine stops angiogenesis directly and indirectly, it can be effective on bone growth [19]. Previous studies have shown that morphine passes through the placenta gradually but leaves the blood circulation very quickly and spreads into tissues such as lung, liver, kidney, spleen, brain and particularly adipose tissue, and in addition to reducing the weight and diameter of the placenta, it also decreases the length and weight of the fetus. The teratogenic effects of morphine in rats happen mostly in the second week of infant development [18].

## Conclusion

The present study confirmed the harmful effects of prenatal morphine consumption on bone formation and bone length, represented in decreased growth plate thickness, cell number in growth plates and deficient cartilage and bone matrix formation. These observations suggest that morphine dependence in pregnant mothers may impair intra-cartilaginous osteogenesis in rat infants. Based on our results the molecular mechanism behind the adverse effects of morphine could be through down regulation of key proteins in cartilage and bone development including SOX9 (as early marker in chondrogenesis), COLL2 (as late marker in chondrogenesis), ALP (as early marker in osteogenesis) and osteocalcin (as late marker in osteogenesis).

## Supplementary Information

**Additional file 1.**

## Data Availability

The datasets used and/or analysed during the current study are available from the corresponding author on reasonable request.

## Abbreviations

H&E: Hematoxyline and Eosin
OD: Optical density
GAG: Glycosaminoglycans
IHC: Immuno-histochemistry
ALP: Alkaline phosphatase
OCN: Osteocalcin
COLL2: Collagen type 2
SOX9: (SRY-Box Transcription Factor 9)
